# Cell-Laden 3D Hydrogels of Type I Collagen Incorporating Bacterial Nanocellulose Fibers

**DOI:** 10.1021/acsabm.3c00126

**Published:** 2023-09-05

**Authors:** Nanthilde Malandain, Hector Sanz-Fraile, Ramon Farré, Jorge Otero, Anna Roig, Anna Laromaine

**Affiliations:** † Institut de Ciència de Materials de Barcelona (ICMAB-CSIC), Campus UAB, 08193 Bellaterra, Spain; ‡ Unitat de Biofísica i Bioenginyeria, Facultat de Medicina i Ciències de la Salut, Universitat de Barcelona, 08036 Barcelona, Spain; § CIBER de Enfermedades Respiratorias, 28029 Madrid, Spain; ∥ Institut d’Investigacions Biomèdiques August Pi i Sunyer, 08036 Barcelona, Spain; ⊥ The Institute for Bioengineering of Catalonia (IBEC), The Barcelona Institute of Science and Technology (BIST), 08028 Barcelona, Spain

**Keywords:** tissue engineering, collagen, bacterial cellulose, composite hydrogels, 3D cell culture

## Abstract

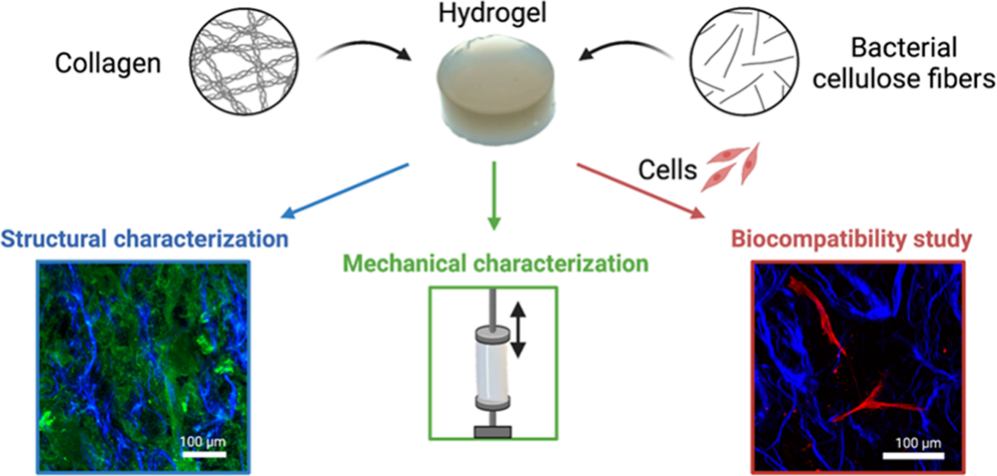

There is a growing interest in developing natural hydrogel-based scaffolds to culture cells in a three-dimensional (3D) millieu that better mimics the *in vivo* cells’ microenvironment. A promising approach is to use hydrogels from animal tissues, such as decellularized extracellular matrices; however, they usually exhibit suboptimal mechanical properties compared to native tissue and their composition with hundreds of different protein complicates to elucidate which stimulus triggers cell’s responses. As simpler scaffolds, type I collagen hydrogels are used to study cell behavior in mechanobiology even though they are also softer than native tissues. In this work, type I collagen is mixed with bacterial nanocellulose fibers (BCf) to develop reinforced scaffolds with mechanical properties suitable for 3D cell culture. BCf were produced from blended pellicles biosynthesized from *Komagataeibacter xylinus*. Then, BCf were mixed with concentrated collagen from rat-tail tendons to form composite hydrogels. Confocal laser scanning microscopy and scanning electron microscopy images confirmed the homogeneous macro- and microdistribution of both natural polymers. Porosity analysis confirmed that BCf do not disrupt the scaffold structure. Tensile strength and rheology measurements demonstrated the reinforcement action of BCf (43% increased stiffness) compared to the collagen hydrogel while maintaining the same viscoelastic response. Additionally, this reinforcement of collagen hydrogels with BCf offers the possibility to mix cells before gelation and then proceed to the culture of the 3D cell scaffolds. We obtained scaffolds with human bone marrow-derived mesenchymal stromal cells or human fibroblasts within the composite hydrogels, allowing a homogeneous 3D viable culture for at least 7 days. A smaller surface shrinkage in the reinforced hydrogels compared to type I collagen hydrogels confirmed the strengthening of the composite hydrogels. These collagen hydrogels reinforced with BCf might emerge as a promising platform for 3D *in vitro* organ modeling, tissue-engineering applications, and suitable to conduct fundamental mechanobiology studies.

## Introduction

Scaffolds affording three-dimensional (3D) cell culture are being exploited in tissue engineering (TE) and regenerative medicine to evaluate novel materials as tissue and organ replacements, and as *in vitro* platforms to study mechanobiology responses.^1^ The characteristics of these 3D scaffolds encompass biocompatibility and interconnected pores to recreate the structure and physical properties of the native tissues’ extracellular matrix (ECM).^2^ Biomaterial-based hydrogels fulfill most of those properties, and additionally, they can be produced from ECM proteins after decellularization of tissues (dECM), thus providing a similar chemical composition to targeted organs, which permits studying cell response in a physiomimetic environment although is difficult to elucidate which proteins are involved in different cellular mechanisms.^3−5^ Thus, for mechanobiology studies, controlled-composition hydrogels are better-suited to answer questions about the role and effect of different ECM proteins. Among those ECM hydrogels, type I collagen extracted from rat-tail tendons^6^ is considered one of the gold standards as collagen modulates cell function and is the main protein component in many tissues.^7,8^ However, one of the limitations of collagen^8^ as well as of dECM^9^ hydrogels is their low mechanical properties compared to native tissues, knowing that the mechanical responses are critical to regulating cell behavior, such as phenotype,^10−12^ proliferation, and spreading.^13,14^ For instance, the differentiation of mesenchymal stromal cells (MSCs) in osteoblasts strongly correlates with the stiffness of their growth substrate.^10−12^ Thus, mechanical properties of the cell microenvironment, whether within the body or in an *in vitro* setting, must be in the appropriate range, calling for stiffer biomimetic dECM hydrogels to recreate native tissues better. The latter demands a scaffold with tuned stiffness of the cell’s microenvironment while not affecting other parameters, such as their microstructure, porosity, or the number of cellular adhesion points. Such stringent characteristics are highly sought-after in mechanobiology to better understand mechanotransduction signaling pathways.^15^

Different strategies have been explored to modify the mechanical properties of dECM hydrogels, such as increasing the solubilized ECM concentration or adding chemical cross-linkers.^8,16^ However, those approaches also affect the structure of the scaffolds and the cell response. For example, an increase in collagen concentration is known to induce malignant phenotypes,^17^ and the addition of some cross-linkers, such as genipin^18,19^ or ultraviolet photoinitiators,^20^ can be toxic and damage the cultured cells. Other strategies are based on the addition of reinforcing materials, such as micron-sized natural silk fibers; unfortunately, this affects the hydrogel’s structure and may alter cell behavior and response.^21^ Without a gold standard method, it is still challenging to create scaffolds that can accommodate cells with improved mechanics and similar concentrations of type I collagen as found in tissues.

Following the latter approach, we evaluated the incorporation of bacterial nanocellulose (BC) fibers in collagen type I hydrogels obtained from rat-tail tendons. BC is a pure natural polymer biosynthesized by bacteria, such as *Komagataeibacter xylinus*.^22^ Produced BC films are flexible, conformable, mechanically stable,^23−26^ and chemically modifiable^27,28^ and could be obtained at a large scale by controlled fermentation. From a biomedical point of view, BC is an endotoxin-free,^27,29^ biocompatible, and suturable^24^ material approved by the Food and Drug Administration (FDA),^30^ such as Biofill, which is the first BC-based medical product commercialized in the 1980s as a skin substitute for wound healing.^31^

Some collagen-based/BC composites have already been prepared following different methods while targeting diverse final uses such as in biomedical applications,^32−36^ tissue regeneration,^37,38^ or tissue-engineered scaffolds.^39−41^ For instance, collagen has already been incorporated into BC films by immersion,^34−36,39,42^ which can be mediated by Fmoc-glycine (Fmoc-Gly) esterification,^37^ by adding collagen to the bacterial culture medium,^40^ or by diffusion under an electric field.^43^ The evaluation of BC films incorporating other biomaterials (hyaluronic acid,^44^ silk^45^) indicates increased tensile strength. However, the porosity range of pure BC films does not allow them to be used as scaffolds for 3D cell culture. Similarly, BC nanofibers (BCf) were used to reinforce photocurable hydrogels^46^ and natural polymers (collagen,^33,41^ ECM,^38^ etc.) without greatly affecting their structure. For instance, a previous study used BCf in collagen hydrogels for bone TE scaffolds to promote the osteogenic differentiation of MSCs.^41^ However, the freeze-drying fabrication step of such hydrogels made the 3D cell culture difficult, and the lack of mechanical characterization prevented any comparison with native tissue stiffness.

Here, we cast type I collagen (Col-I), BCf, and cells in a single step creating a tissue-engineered scaffold where cells are cultured in 3D within it, which can prove useful for 3D *in vitro* organ modeling, or fundamental mechanobiology studies. BCf has allowed us to improve the mechanical properties of the hydrogel without the addition of external cross-linkers or affecting the porosity. We evaluated the distribution of the components, mechanical properties of the composite hydrogel, and viability of cells (human bone marrow mesenchymal stromal cells (hBM-MSCs) and human fibroblasts (HLFs)) cultured in the developed 3D composites scaffolds.

## Materials and Methods

### Materials

The bacteria strain *K. xylinus* (NCIMB 5346) was purchased from CECT (Spain). The culture media was prepared using glucose, peptone, yeast extract purchased from Condalab (Spain), Na_2_HPO_4_·12H_2_O, and citrate acid monohydrate from Sigma-Aldrich (Merck, Spain).

Human bone marrow-derived mesenchymal stromal cells (hBM-MSCs) and human lung fibroblasts (HLFs) were purchased from ATCC (PCS-500-012) as well as the culture media containing 125 pg/mL recombinant human fibroblast growth factor (rh FGF), 15 ng/mL recombinant human insulin-like growth factor-1, 7% fetal bovine serum (FBS), and 2.4 mM l-alanyl-l-glutamine. Human lung fibroblasts (HLFs) were purchased from ATCC (PCS-201-013) as well as the culture media containing 7.5 mM l-glutamine, 1 μg/mL hydrocortisone hemisuccinate, 50 μg/mL ascorbic acid, 5 ng/mL rh FGF, 5 μg/mL recombinant human insulin, and 2% FBS.

For all of the experiments, sodium hydroxide (NaOH), acid acetic, and paraformaldehyde (PFA) were purchased from Sigma-Aldrich, acetone, and isopropanol from PanReac AppliChem (Spain), and phosphate-buffered saline (PBS) 1× and 10×, Trypsin, and FBS from Gibco (Fisher Scientific, Spain).

### 3D Culture of Human Bone Marrow-Derived Mesenchymal Stromal Cells (hBM-MSCs) and Human Lung Fibroblasts (HLFs)

hBM-MSCs and HLFs were expanded following the manufacturer’s instructions, and all experiments were performed with cells at passages 3–6 cultured at 37 °C with 5% CO_2_. For cell viability assays, cultured hBM-MSCs or HLFs were trypsinized, resuspended in culture media, and mixed (3 × 10^5^ cells/mL) with the pregels. The culture medium was added once the hydrogels were jellified. The CellTiter-Glo 3D assay (Promega, Spain) was used to quantify the viability of hBM-MSCs at 1, 4, and 7 days of culture, following the manufacturer’s instructions. hBM-MSCs cultured in a conventional dish (2D) were used as a control. For the hydrogel shrinkage experiment, HLFs were mixed (3 × 10^5^ cells/mL) with the pregels and the cell culture medium was added after gelation. After detaching the sides and bottoms, or only the sides of the hydrogels from the well plate to remove adhesions, they were stored at 37 °C for 7 days. Their area was measured from pictures taken on day 7 and the percentage of shrinkage was calculated compared to the area of the acellular scaffolds as reported by Falcones et al.^4^

### Type I Collagen (Col-I)

Rat-tail type I collagen was extracted according to the protocol published by Rajan et al.^47^ In brief, tails from Sprague-Dawley rats from other experiments at the animal facility of the School of Medicine (University of Barcelona) were collected. Tendons were extracted, rinsed successively with PBS, acetone, and isopropanol, and then dissolved in 0.02 N acetic acid for 48 h at 4 °C. The solution was then blended, freeze-dried for 48 h (Telstar Lyoquest-55 Plus), and the resulting sponge was weighed and solubilized in 0.02 N acetic acid at a concentration of 10 mg/mL. The obtained solution was stored at 4 °C for further use. The ethical committee approved all animal care and experimental procedures for animal research at the University of Barcelona.

### Bacterial Nanocellulose Fibers (BCf)

Bacterial nanocellulose (BC) films produced by the *K. xylinus* strain were obtained, as previously reported by Anton-Sales et al.^24^ In brief, 0.5 mL of bacteria were cultured in 4.5 mL of Hestrin–Schramm (HS) medium that is composed of 1.15 g/L citric acid, 6.8 g/L Na_2_HPO_4_·12H_2_O, 5 g/L peptone, 5 g/L yeast extract, and 20 g/L dextrose in Milli-Q water for 7 days at 30 °C. Then, the former bacterial solution in a proportion of 1:14 with HS fresh medium was poured into 12 × 12 cm^2^ plates and cultured for 7 days at 30 °C. As-obtained BC films were soaked in a solution of 1:1 ethanol/Milli-Q water for 10 min to kill bacteria. The cleaning process to remove all organic residues involved 20 min in Milli-Q boiling water and two periods of 20 min in NaOH 0.1 M. Finally, BC films were rinsed in Milli-Q water until reaching the neutral pH (pH = 7.4) and autoclaved (121 °C, 20 min).

As previously reported by Roig-Sanchez et al.,^46^ bacterial nanocellulose fibers (BCf) were obtained after blending five films of 144 cm^2^ in 1 L of Milli-Q water with a commercial household blender (Jata electro Mod. BT1200, maximum speed, 10 min). The solution was then filtered with a Stericup Quick Release Filter Millipore with a PES membrane of 0.22 μm in a laminar flow cabinet. BCf aggregates were collected from the filter and autoclaved again (121 °C, 20 min).^48^ The water content of BCf aggregates was determined by weighing 2 g of BCf aggregates before and after drying at 60 °C. BCf were then placed in suspension in PBS 1× at 12 mg/mL solid content. BCf length and width are 14 ± 9 μm and 42 ± 7 nm, respectively.

### Endotoxin Evaluation

The endotoxin level was quantified using the Pierce Chromogenic Endotoxin Quant Kit (ThermoFisher, Spain) after incubating BCf in 40 mL of endotoxin-free water at 37 °C for 72 h, which respects the FDA regulation.

We obtained 32 ± 3 EU per gram of solid content of BCf, corresponding to 0.024 EU per hydrogel, as one hydrogel contains 0.75 mg of BCf. This level is considerably below the FDA limit of 20 EU/device for medical devices that will not be in contact with the cerebrospinal fluid.^49^

### BCf Decorated with Superparamagnetic Iron Oxide Nanoparticles (BCf-SPIONs)

BCf-SPIONs were prepared as previously reported by Mira-Cuenca et al.^48^ Briefly, 5 g of BCf were solvent-exchanged in benzyl alcohol overnight. Then BCf were mixed with 40 mL of benzyl alcohol and 3.11 mmol (1100 mg) of Fe(acac)_3_ in a microwave tube. The dispersion was heated for 5 min at 60 °C (300 W) and 10 min at 210 °C (750 W) in a microwave oven with a controlled atmosphere (Advanced flexible microwave synthesis platform from Millestone) operating at a frequency of 2.45 GHz and a maximum power of 750 W and 5% agitation. At the end of the reaction, the solution was cooled to 50 °C, filtered, and cleaned twice with acetone and once in Milli-Q water. SPIONs produced were grafted to the BCf, which was seen in the change in color from white to brown. Then, BCf-SPIONs were autoclaved in Milli-Q water at 121 °C for 20 min and stored at room temperature for further use.

### Col-I/BCf Hydrogel Scaffold Development

To allow the gelation, type I collagen pregel was prepared by adjusting the pH to neutral (pH = 7.4) with cold NaOH 1 M and the salt concentration to the physiological one with PBS 10× at 4 °C. The pregel was then mixed with BCf suspension at 4 °C and centrifuged at 340*g* for 2 min to pull air bubbles that can be formed during mixing out of it, and no sedimentation of the fibers was observed. Finally, the pregel was dropped on a 24-well plate and left to jellify in an incubator for 30 min at 37 °C. The hydrogels were composed of a solid content corresponding to 74% (0.70% w/w) Col-I and 26% (0.25% w/w) BCf and will be referred to as Col-I/BCf. BCf-SPIONs were mixed with collagen and will be referred to as Col-I/BCf-SPIONs. As a control, collagen hydrogels were prepared without the addition of BCf, to obtain a 7 mg/mL concentration (solid content: 100% Col-I) and are referred to as Col-I.

### Gelation Time

Col-I and Col-I/BCf hydrogels’ gelation times were measured using a rheometer (HAAKE RheoStress 1, ThermoFisher) with 35 mm serrated parallel plate geometry. Pregels were placed on a Peltier plate set at 4 °C and performed for 10 min with a 0.2 mm gap, 0.1 Hz frequency, and 0.5% strain. The temperature was then increased to 37 °C to induce the gelation while maintaining all of the other settings for 10 min.

### Porosity Analysis by Adsorption and Desorption of Nitrogen

The total porosity of the scaffolds was calculated from the density of the materials according to eq 1
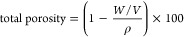
1where *W* is the dried hydrogel weight, *V* is the hydrogel’s volume, and ρ is the density of the polymers. An estimated porosity range was calculated with high and low densities of Col-I and BCf of 0.89–1.34 g/cm^3^ ^51,50^ and 1.25–1.61 g/cm^3^, ^53,52^ respectively. The volume of dried scaffolds was considered with 10% shrinkage due to the solvent exchange phase compared to the wet form.^54^

The volume and diameter of the mesopores of the scaffolds were measured by adsorption and desorption of nitrogen using BET and BJH methods after the supercritical drying of the hydrogels. In brief, Col-I and Col-I/BCf hydrogels were fixed for 60 h in 4% PFA in PBS 1× and then washed with 0.2 M phosphate buffer for 24 h. After washing with Milli-Q water, hydrogels had a solvent exchange phase with ethanol solutions with increasing concentrations from 50% to absolute ethanol at 4 °C and were loaded into a high-pressure autoclave vessel (300 mL) filled with pure ethanol for supercritical drying. First, the autoclave was pressurized to 100 bars at room temperature. Second, ethanol was exchanged for liquid CO_2_ at a flow rate of 1.5 kg/h for 2 h. To reach the supercritical state of CO_2_, the temperature and pressure must be higher than 31 °C and 74 bars, respectively. Thus, while keeping the liquid CO_2_ at a flow rate of 1.5 kg/h, the reactor was heated to 45 °C for 1 h to transform the liquid CO_2_ into the supercritical state and remove unreacted species. This supercritical state was kept for 3 h by keeping the same parameters and stopping the CO_2_ flow. Finally, the autoclave was slowly depressurized, and the dry hydrogels were removed. Nitrogen adsorption/desorption measurements were conducted by a particle size analyzer (ASAP 2000 V2.04; Micromeritics) after degassing for 20 h at 60 °C.

The proportion of macropores was calculated from the total porosities and volume of mesopores (adsorption).

### Scanning Electron Microscopy (SEM)

The microstructures of the supercritically dried Col-I and Col-I/BCf scaffolds (detailed in the Porosity Analysis by Adsorption and Desorption of Nitrogen section) were characterized using SEM. Samples were placed on a SEM aluminum substrate over a carbon tape adhesive and imaged with a scanning electron microscope (QUANTA FEI 200 (FEG)) at 15 kV under vacuum (60 Pa).

### Assessment of Mechanical Properties by Tensile Test

The tensile stretch test measured the mechanical properties of Col-I and Col-I/BCf hydrogels in cylinder shapes (5 × 2 mm^2^). Each end of the sample was glued with cyanoacrylate to small hooks. One hook was attached to the lever of a servo-controlled displacement actuator with an integrated force sensor (300C-LR; Aurora Scientific, Canada), and the other hook was fixed to stretch the hydrogel while measuring the extended length and applied force. As described by Farré et al., the stress (σ) and elastic modulus (*E*_M_) at 20% strain were obtained from a series of 10 force–displacement curves at 0.2 Hz frequency and 30% maximum strain.^55^

### Measurement of Rheological Properties

The properties of Col-I and Col-I/BCf hydrogels were measured by using a rheometer (HAAKE RheoStress 1; ThermoFisher) with 35 mm serrated parallel plate geometry. Hydrogel cylinders (2.1 cm in diameter and 2 mm in height) were prepared and stored overnight at 4 °C in PBS 1×. The hydrogel samples were placed on a Peltier plate set at 37 °C with a 1 mm gap. To choose a frequency in the range of the viscoelastic region, a trial was performed at constant strain (0.5%) over a frequency range of 0.1–100 Hz while measuring the storage (*G*′) and loss (*G*″) moduli and the modulus of the complex viscosity (μ). Thus, a frequency of 0.5 Hz was chosen for the following experiments. Hydrogels were then analyzed at a constant temperature (37 °C) and frequency (0.5 Hz) over a strain range of 0.1–1000%. The end of the linear viscoelastic region for hydrogels can be found when the storage modulus decreases by 5% from its initial value, being 1.9% for Col-I hydrogels and 1.1% for Col-I/BCf hydrogels. Given these results, a strain of 0.5% was chosen for the following experiments where *G*′, *G*″, and μ were calculated for each sample as the mean of two values measured in the linear viscoelastic region (a strain of 0.5 ± 0.15%).

### BCf Distribution in the Scaffolds Assessed by Confocal Laser Scanning Microscopy

To evaluate the homogeneous dispersion of BCf in the Col-I/BCf hydrogels at the microscale, both materials were stained and imaged by a confocal laser scanning microscope (TI-HUBC, Nikon) with a 20x objective. Hydrogels were fixed with PFA 4% in PBS 1× for 20 min. After repeated washes, they were blocked with 10% FBS for 45 min and incubated with anti-collagen-I rabbit polyclonal (1/300, ab34710, Abcam) for 1 h at 37 °C. Samples were then incubated with the secondary antibody Alexa Fluor 488 anti-rabbit (1/200, ab150061, Abcam) for 1 h and with Calcofluor white (1 mg/mL in PBS 1×) for 10 min, followed by repeated washes to remove the unreacted species. Z-stacks obtained were used to evaluate the 3D spatial distribution of BCf within the structures.

### Viability Analysis of Cells Cultured Within the Hydrogels by Confocal Laser Scanning Microscopy

Live imaging of the cells confirmed cell viability within the hydrogels after 1, 4, and 7 days of 3D culture within the scaffolds. Cells were stained with a live/dead viability kit (Thermofisher) following the manufacturer’s instructions. Thus, hydrogels were washed with PBS 1× and incubated with calcein-AM (live cells, green) and ethidium homodimer-1 (dead cells, red) for 40 min at 37 °C. Samples were then rewashed with PBS 1× before being immediately imaged with a confocal laser scanning microscope (TI-HUBC, Nikon) with a 20× objective. Z-stacks obtained were used to evaluate the 3D spatial distribution of cells within the structures.

### Statistical Analysis

Data are expressed as mean ± standard deviation. Statistical analyses were performed with Graph Pad Prism 9 software using the Shapiro–Wilk normality test followed by an unpaired parametric *t*-test, a one-way ANOVA, or a Kruskal–Wallis test, depending on the conditions. Statistical significance was considered for *p*-values ≤ 0.05 and summarized as *p ≤ 0.05, **p ≤ 0.01, ***p ≤ 0.001, and ****p ≤ 0.0001.

## Results and Discussion

**Figure 1:**
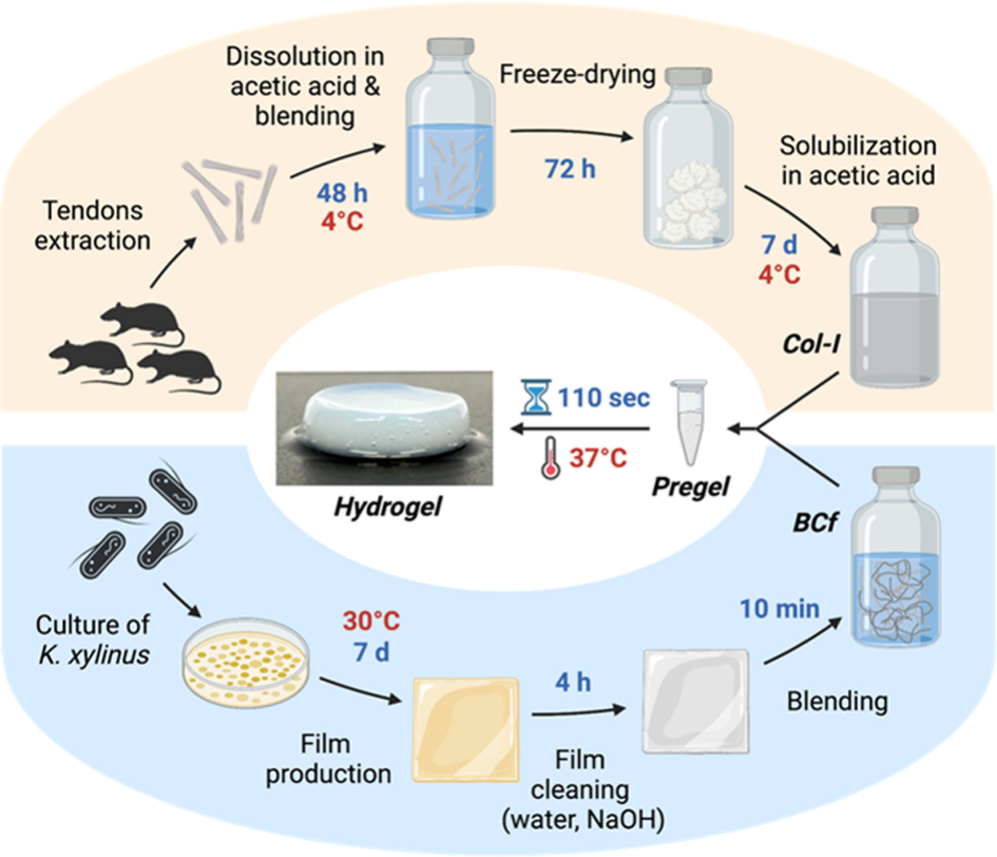
Preparation protocol of Col-I/BCf hydrogels. Col-I was extracted from rat-tail tendons (yellow background), while BCf were produced from BC films derived from the culture of *K. xylinus* (blue background). After mixing both biomaterials, the Col-I/BCf pregel was obtained forming a hydrogel after 110 s at 37 °C.

Composite hydrogels were prepared by mixing bacterial nanocellulose fibers (BCf) obtained from blended BC films and type I collagen (Col-I) obtained from rat-tail tendons (see details in the Material and Methods section and Figure 1). The mixture (pregel) jellified within 2 min on heated plates (37 °C), resulting in a consistent 3D hydrogel.

### Hydrogel Preparation

**Figure 2:**
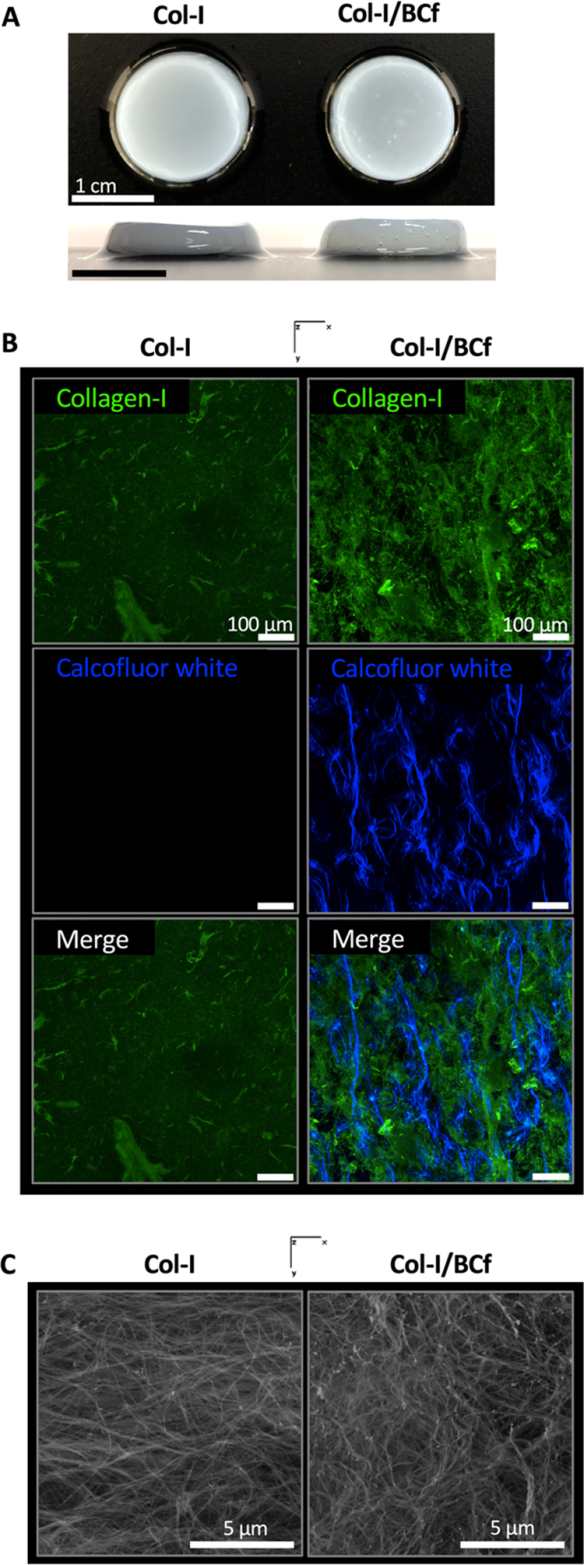
Distribution of Col-I and BCf within the hydrogel. (A) Digital images of Col-I and Col-I/BCf hydrogels. Scale bars: 1 cm. (B) Maximum intensity projection images of 50 μm Z-stacks of confocal microscope images of Col-I and Col-I/BCf hydrogels where collagen-I is stained by indirect immunofluorescence (green), and BCf by Calcofluor white (blue). Scale bar: 100 μm. (C) Representative SEM images of cross sections of Col-I and Col-I/BCf hydrogels.

Several compositions with different ratios of Col-I and BCf were tested (Table 1). The results showed that it was possible to decrease the amount of collagen to 50% solid content and still obtain a gel without using additional cross-linkers. Preparations having a concentration of collagen below 5 mg/mL or with a solid content of BCf higher than Col-I did not gel. Composite hydrogels with enhanced mechanical properties while maintaining the easiness of manipulation were only ensured by using high collagen concentrations (>5 mg/mL). Therefore, the composite with 74% Col-I and 26% BCf (Col-I/BCf) was selected for further studies and compared with the 100% collagen (Col-I), since it could be easily handled while still containing a high proportion of BCf (gray background, Table 1). This Col-I/BCf hydrogel exhibited similar color and transparency to the 100% collagen one, while seeming qualitatively more robust (Figure 2A). In fact, the cylindrical gel from the Col-I/BCf composite has straighter verticality than the Col-I hydrogel, which appears more collapsed (lateral view picture, Figures 2A and S1). The impact of BCf on the gelation time was assessed by subjecting the pregel from 4 to 37 °C to induce the gelation while measuring the rheological properties. No significant differences in the Col-I/BCf hydrogel (103 ± 15 s) were observed compared to the collagen control Col-I (118 ± 8 s). Stability in PBS at room temperature was monitored, and after 4 months, both hydrogels showed highly conserved macrostructures and could still be easily manipulated.

**Table 1:**
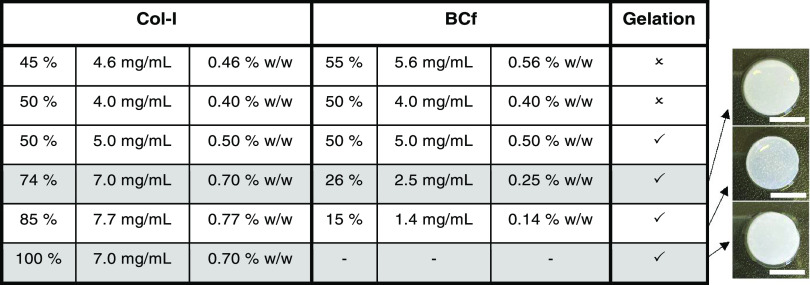
Proportions of Type I Collagen and BCf in Hydrogels to Allow Gelation^a^

a Percentages of solid content with respect to the hydrogel’s total solid content (%) and its total weight (% w/w). Digital images of jellified hydrogels from the top after incubation at 37 °C. Scale bar: 1 cm.

### Macro- and Microstructure of Col-I/BCf Hydrogels

BCf distribution within the hydrogels showed to be uniform without visible fiber aggregation (Figure 2A) that has been confirmed using colored BCf that were decorated by iron oxide nanoparticles (SPIONs) (Figure S1). To corroborate this observation at the microscopic scale, both materials were stained and observed by confocal laser scanning microscopy, which validated their homogeneous distribution (Figure 2B). Moreover, scanning electron microscopy (SEM) established that no phase separation between the two components occurred even after supercritical drying (Figure 2C). Therefore, due to its nanosize, the presence of BCf did not affect the gelation of the hydrogels nor the fiber organization; thus, from the characterization experiments conducted, we can state that by using the developed protocol with an adequate ratio of the two components, Col-I and BCf were homogeneously mixed.

The porosity of the hydrogels, which can affect cell growth, was determined by N_2_ adsorption (Figure S2). The obtained isotherms of Col-I/BCf and Col-I are characteristic of type II with hysteresis similar to the H3 loop corresponding to macroporous materials (Figure S2A,B).^56^ The pore size distribution was similar in both hydrogel types, but Col-I/BCf showed a larger surface area than the Col-I scaffold (Table S1). The most prevalent pore diameter was slightly lower in Col-I/BCf (39 nm) than in Col-I hydrogels (53 nm) (Figure S2C and Table S1). In addition, the calculated total porosity and the macropore proportion were 99.1 ± 0.3 and 99.8 ± 0.01% for Col-I hydrogel, and 99.5 ± 0.1 and 99.7 ± 0.1% in the presence of BCf. Thus, adding BCf does not significantly change the hydrogel’s nanostructure while ensuring a proper porosity for cell growth.

### Mechanical Properties of Col-I/BCf Hydrogels

**Figure 3:**
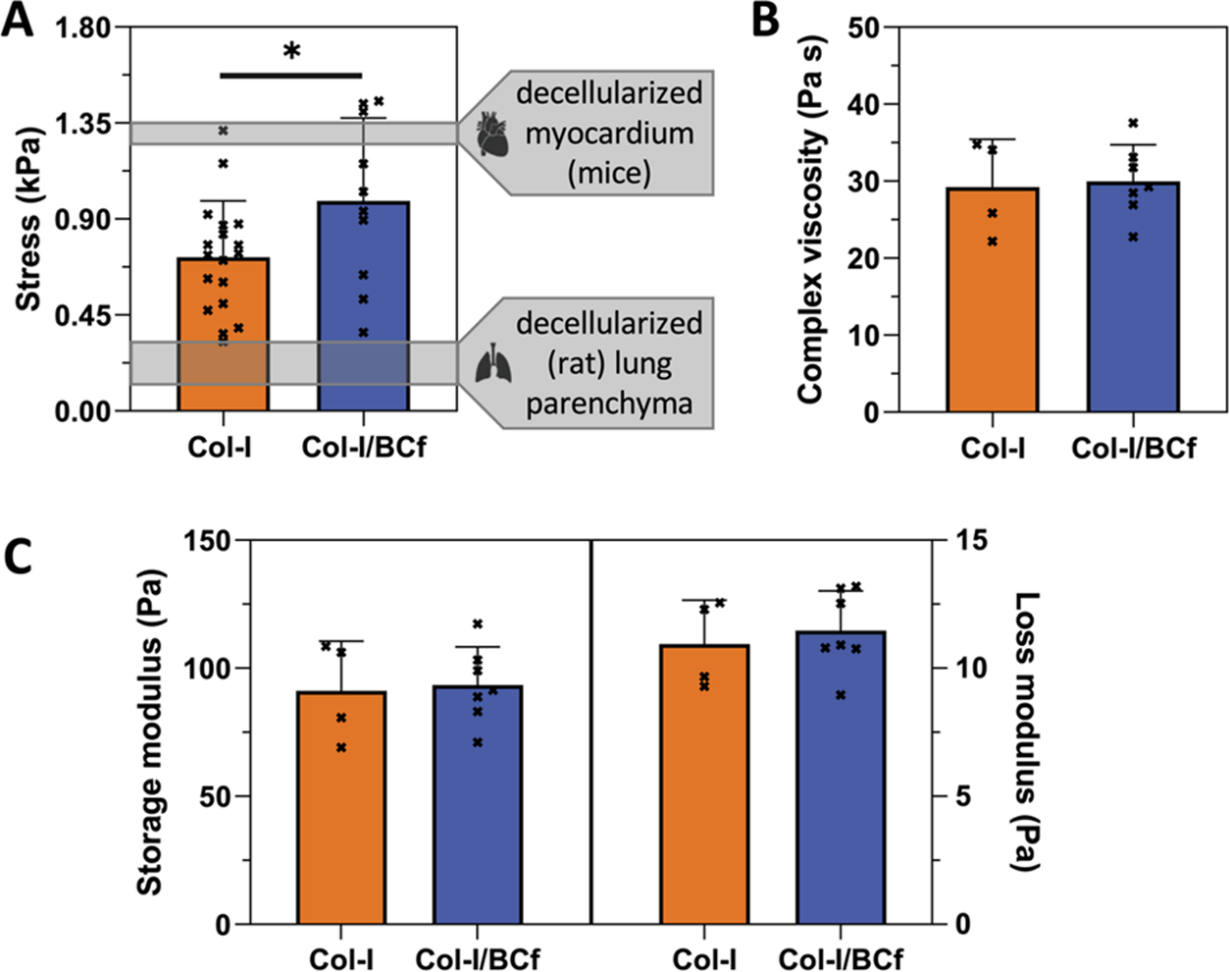
Mechanical properties of hydrogels. (A) Stress measured by a tensile test at 20% strain (*n* = 18 for Col-I and *n* = 10 for Col-I/BCf). *p ≤ 0.05 according to the unpaired *t*-test. (B, C) Rheological nonstatistically significant data measured at 0.5% strain, 0.5 Hz frequency, and 37 °C (*n* = 4 for Col-I and *n* = 7 for Col-I/BCf).

Macroscale mechanics of Col-I and Col-I/BCf hydrogels were studied by uniaxial tensile testing. The stress was calculated at a selected strain of 20%, which is slightly higher than physiologically cardiac (10–12%) and respiratory (12–20%) tissues,^57^ and it is commonly used for the characterization of this kind of scaffolds.^55^ Col-I hydrogels showed a stress value of 0.7 ± 0.3 kPa, while Col-I/BCf was 43% higher (σ = 1.0 ± 0.4 kPa) (Figure 3A). Those values of Col-I hydrogel were similar to the data reported by Sanz-Fraile et al. using the same equipment.^21^ Moreover, the values obtained for Col-I/BCf hydrogels were similar to reported scaffolds of collagen and silk (1:1). However, in the case of the latter scaffolds, the hydrogel structure was affected by the addition of micron-sized silk fibers.^21^ If we compare the mechanical properties (measured with the same equipment) of Col-I/BCf hydrogels with rat decellularized lung parenchyma or mice decellularized ventricular myocardium, we found that Col-I/BCf hydrogel is stiffer than lung tissue (0.2–0.3 kPa)^58^ and similar to the ventricular myocardium stiffness (σ = 1.24–1.35 kPa).^55^

Storage modulus (*G*′), loss modulus (*G*″), and complex viscosity (μ) of Col-I and Col-I/BCf hydrogels were measured by rheometry at 0.5% strain, 0.5 Hz frequency, and 37 °C. As shown in Figure 3B,C, Col-I and Col-I/BCf hydrogels displayed similar values for each parameter (*G*′_Col-I_ = 91 ± 19 Pa, *G*″_Col-I_ = 11 ± 2 Pa, μ_Col-I_ = 29 ± 6 Pa·s; *G*′_Col-I/BCf_ = 93 ± 15 Pa, *G*″_Col-I/BCf_ = 12 ± 2 Pa, μ_Col-I/BCf_ = 30 ± 5 Pa·s). Therefore, the presence of BCf did not affect the viscoelastic properties of the hydrogel, keeping it in the same range of pure Col-I hydrogels that have been extensively shown to be suitable in 3D printing techniques.^21,59^

### Viability of hBM-MSCs 3D Cultured in Col-I/BCf Scaffolds

**Figure 4:**
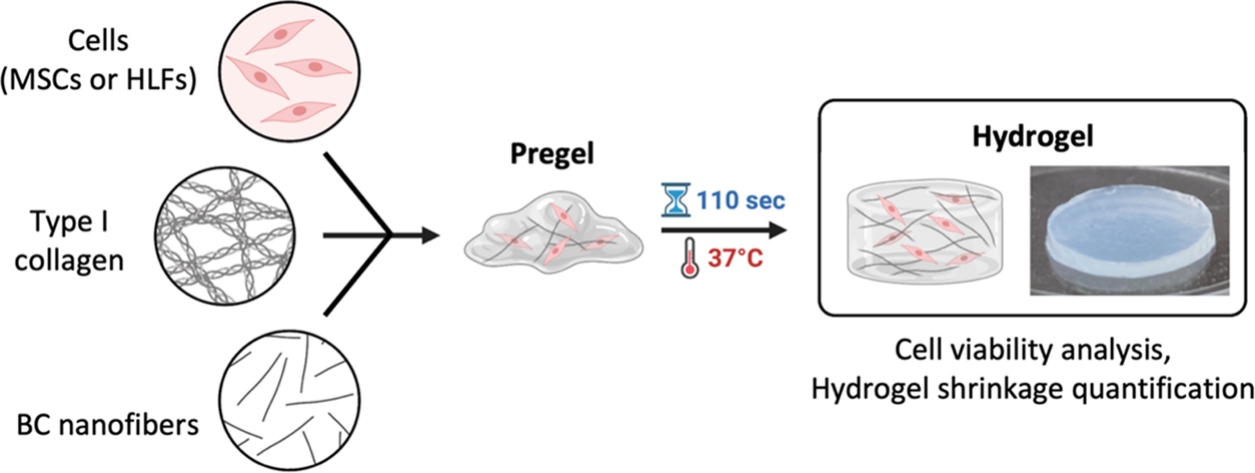
3D culture of cells within the hydrogels. Type I collagen, BC nanofibers, and cells were mixed to obtain a pregel that was then incubated at 37 °C to jellify. Cells were cultured for 7 days to evaluate the cell viability and hydrogel shrinkage. Scale bar: 1 cm.

**Figure 5:**
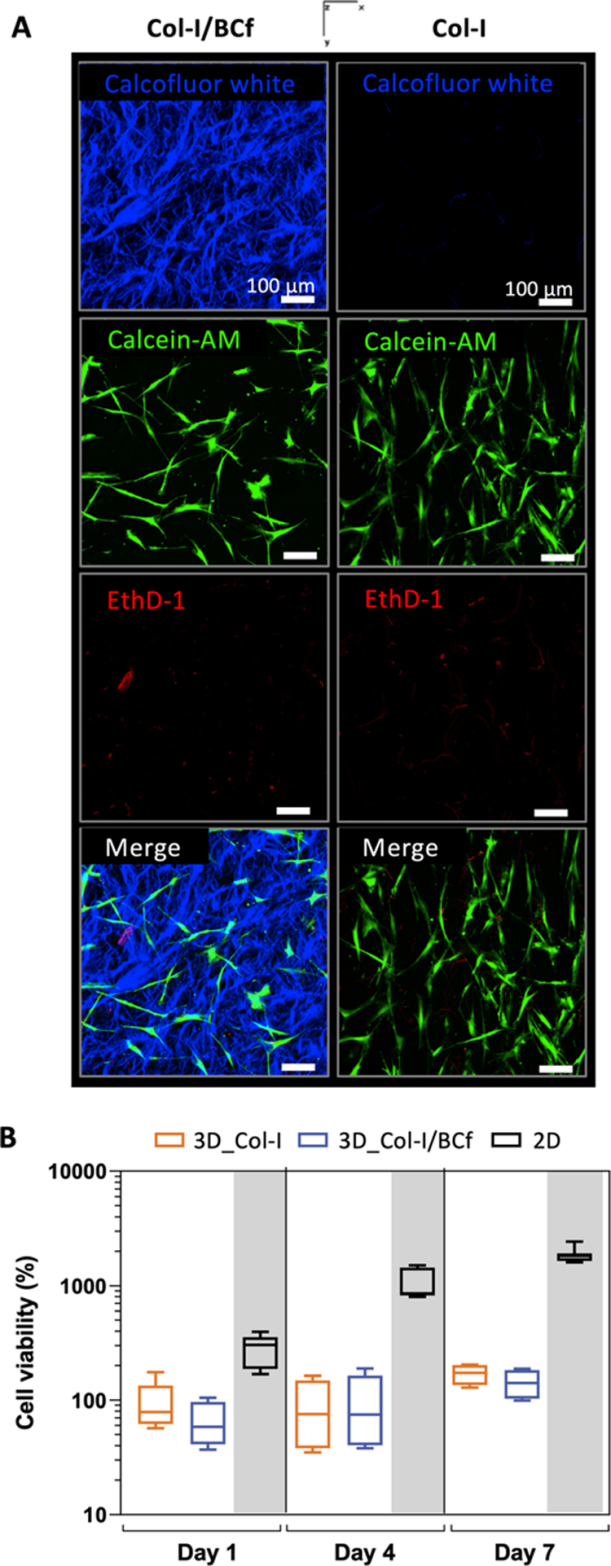
3D culture of human bone marrow-derived mesenchymal stromal cells (hBM-MSCs) within the hydrogels. (A) Maximum intensity projection images of 200 μm Z-stacks of live confocal images of stained hBM-MSCs after being cultured within the hydrogels for 7 days. hBM-MSCs were stained with a live/dead kit where live cells appear in green (Calcein-AM) and dead cells in red (Ethidium homodimer-1, EthD-1). Calcofluor white has been used to stain BCf in blue. (B) Cell viability of hBM-MSCs quantified after 1 day (*n* = 3), 4 days (*n* = 3), and 7 (*n* = 2) days of culture in 3D in Col-I (orange) and Col-I/BCf (blue) hydrogels and in 2D on conventional culture plastic (black). White and gray backgrounds correspond to the culture of hBM-MSCs in hydrogels and on plastic-2D (control, Ctrl). No statistical significance was found between each condition with the Kruskal–Wallis nonparametric test.

As mentioned, many 3D scaffolds are produced by using cross-linkers, toxic chemicals, or high temperatures during the gelation process. Hence, cells cannot be incorporated in this step, which complicates obtaining homogeneous 3D cell distribution within the scaffold. In this work, the gelation process did not require an additional cross-linker, so in a single step, the scaffold was produced, and the cells were incorporated inside (Figure 4). Human bone marrow-derived mesenchymal stromal cells (hBM-MSCs) were chosen since they are mechanosensitive adult stem cells widely used in TE applications and mechanobiology studies for their regenerative potential. Col-I/BCf pregel and cells were mixed at an initial concentration of 3 × 10^5^ cells/mL. Pregels were formed and incubated at 37 °C to obtain hydrogels with a cylindrical shape of 0.16 cm in height. Once jellified, the culture medium was added, and the cells were cultured for 7 days within the scaffolds. The distribution and viability of hBM-MSCs were then evaluated. Cell distribution was homogeneous in the volume of scaffolds independent of the presence or absence of BCf, as imaged by confocal microscopy (Figure 5A). However, the cell density could be optimized in future work to obtain a more realistic tissue-like structure. To compare the cell viability and proliferation within both scaffolds in detail, the ATP produced was quantified after 1, 4, and 7 days of culture. The number of alive cells is constant during the 7 days of culture in both hydrogels, as shown in Figure 5B. As expected, cell proliferation was higher in the 2D conventional dish than in 3D, which is coherent with data reported in previous studies.^60−62^ Thus, the presence of BCf, increased stiffness, and slight porosity change of Col-I/BCf hydrogel compared to Col-I do not affect cell viability and growth, making it a suitable scaffold for 3D stromal cell culture.

### Shrinkage of Col-I/BCf Scaffolds during the 3D Culture of HLFs

**Figure 6:**
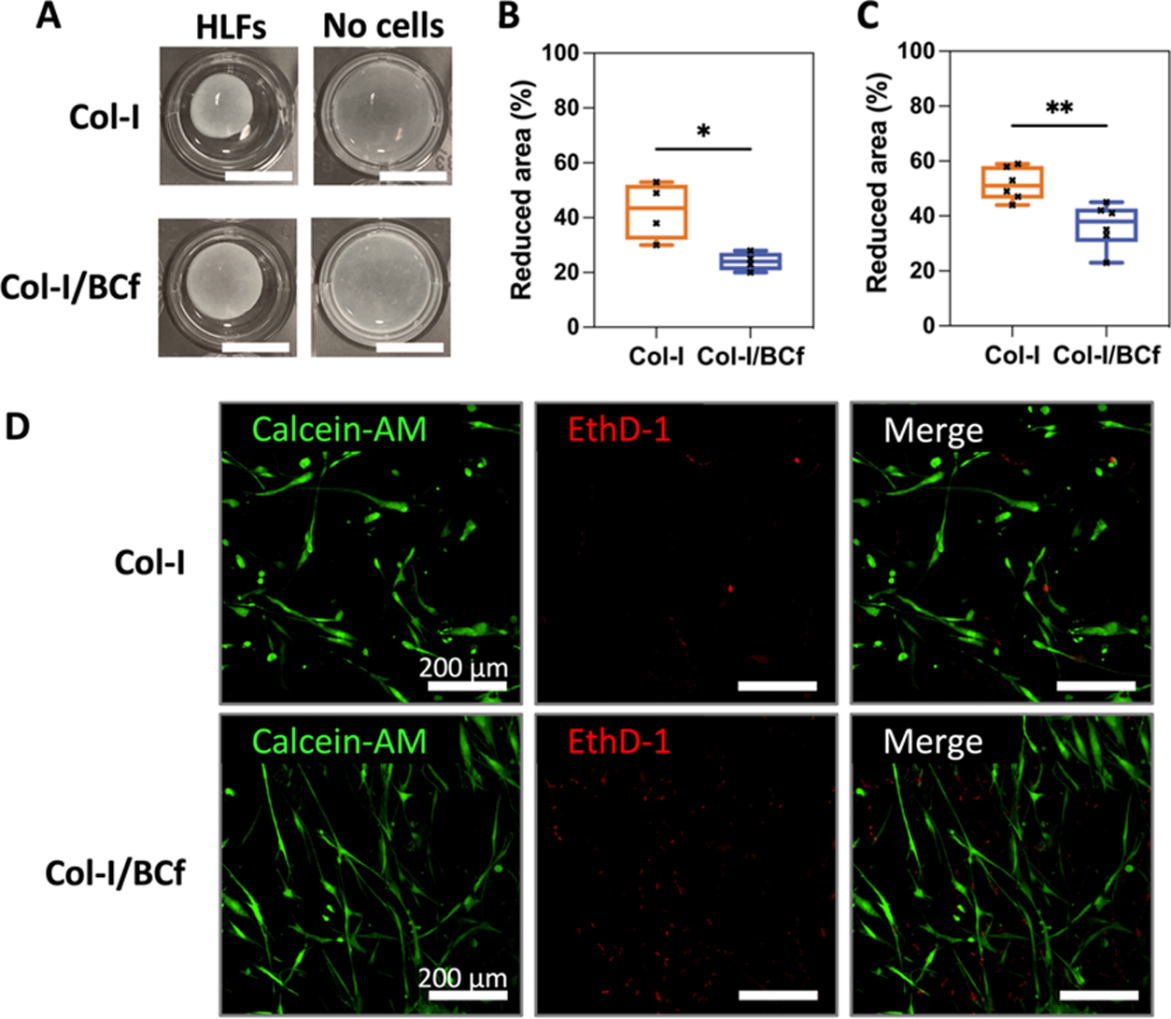
Shrinkage of the hydrogels with 3D cultured human lung fibroblasts (HLFs). (A) Pictures of Col-I and Col-I/BCf hydrogels completely detached (SB) with and without HLFs. Scale bars: 1 cm. (B, C) Quantification of the reduced area of hydrogels containing HLFs compared to acellular hydrogels while having detached only the sides (B), or sides and bottoms (C) of hydrogels after 7 days of 3D culture. (D) Maximum intensity projection images of 150 μm Z-stacks of live confocal images of stained HLFs after being cultured within the hydrogels for 7 days. Cells were stained with a live/dead kit where live cells appear in green (Calcein-AM) and dead cells in red (EthD-1). *p ≤ 0.05 and **p ≤ 0.01.

Fibroblasts are adherent cells highly studied in different fields due to their high production rate of ECM and their interactions with it.^63^ It is known that these cells can remodel the matrix and induce shrinkage of soft collagen hydrogels depending on the cell density and collagen concentration.^64^ The mechanism of this phenomenon is still unclear; however, it is suggested that collagen contraction is due to its degradation carried out by various proteins such as matrix metalloproteinases^65^ or to cellular forces applied to matrix fibers.^66^ HLFs were mixed with pregels and incubated at 37 °C to induce the gelation before adding the cell medium to study the cell viability of an adherent cell type and confirm the reinforcement of the hydrogels. Hydrogels’ sides and bottoms (SB) were detached from the plastic well plate as collagen hydrogels adhere to the surface while it jellifies. Some hydrogels’ sides were detached while keeping their adhesion with the bottom (S). HLFs were cultured within the scaffolds for 7 days, and as visible in Figure 6A, the acellular scaffolds did not shrink. Thus, the hydrogel shrinkage was evaluated by quantifying the reduced area compared to these. As expected, completely detached hydrogels (SB) had a higher reduced area than the ones with only detached sides (S) (Figure 6B,C). In both cases, Col-I/BCf hydrogels shrank less than the Col-I ones, with area reductions of 24 ± 3% (S) and 37 ± 8% (SB) against 43 ± 10% (S) and 52 ± 6% (SB), respectively. Thus, in composite scaffolds, BCf acted like a mesh that maintained the hydrogel shape and dimensions, as cells cannot degrade it. Moreover, cell viability was maintained after 7 days of culture, as confirmed by imaging them by laser confocal scanning microscopy after live/dead staining of the scaffolds (Figure 6D).

## Conclusions

Here, we presented Col-I/BCf hydrogels produced in a single step as scaffolds for 3D cell culture. Both natural polymers were homogeneously distributed, and the macro- and microstructures of the composite hydrogel were similar to those of Col-I. The stiffness of the Col-I/BCf scaffold increased by 43% while preserving the suitable viscoelastic properties of collagen. In this study, the increased stiffness achieved with the Col-I/BCf hydrogels is coherent with the fact that type I collagen remains the main component (0.70% w/w). We believe that the possibility of tuning the mechanical properties of type I collagen hydrogels with BCf and without increasing the collagen concentration is interesting.

Additionally, our fabrication method does not include the use of any cross-linker, and cell-laden hydrogel scaffolds could be produced in a single step by mixing type I collagen, BCf, and cells (human bone marrow mesenchymal stromal cells (hBM-MSCs) and human fibroblasts (HLFs)), resulting in homogeneous 3D cell cultures. We cultured MSCs and HLFs in the presence of BCf for 7 days and achieved good viability. Reinforcement was also proved by a smaller hydrogel′s surface shrinkage for the cell-laden reinforced hydrogels. Thus, BCf are useful to reinforce the hydrogel and no adverse effects have been detected due to the nanosize of its fibers. The combination of collagen reinforced with BCf emerges as a potential scaffold for *in vitro* studies where collagen hydrogel with enhanced mechanical properties is required.

## Supporting Information Available

Porosity analysis of the hydrogels including isotherms, pore size distribution histogram, and a table with data obtained from the adsorption and desorption of nitrogen (PDF)
