# Supplementary material for: Cell-Laden 3D Hydrogels of Type I Collagen Incorporating Bacterial Nanocellulose Fibers
Source: ACS Appl Bio Mater. 2023 Sep 5;6(9):3638–47. doi: 10.1021/acsabm.3c00126 (PMC10521014; doi:10.1021/acsabm.3c00126)
Supplement: Supplementary file 1 — mt3c00126_si_001.pdf [file mt3c00126_si_001.pdf]

Supplementary Information

Cell-laden 3D-hydrogels of type I collagen incorporating bacterial nanocellulose fibers

Nanthilde Malandain[1,2], Hector Sanz-Fraile[2], Ramon Farré[2,3,4], Jorge Otero[2,3,5], Anna Roig[1*] and Anna Laromaine[1*]*

[1] Institut de Ciència de Materials de Barcelona (ICMAB-CSIC), Campus UAB, 08193 Bellaterra, Spain.

[2] Unitat de Biofísica i Bioenginyeria, Facultat de Medicina i Ciències de la Salut, Universitat de Barcelona, 08036 Barcelona, Spain.

[3] CIBER de Enfermedades Respiratorias, 28029 Madrid, Spain.

[4] Institut d'Investigacions Biomèdiques August Pi i Sunyer, 08036 Barcelona, Spain.

[5] The Institute for Bioengineering of Catalonia (IBEC), The Barcelona Institute of Science and Technology (BIST), 08028 Barcelona, Spain.

*Corresponding authors: jorge.otero@ub.edu (J.O.); roig@icmab.es (A.R.); alaromaine@icmab.es (A.L.)

Figure S1. Col-I/BCf hydrogels functionalized with iron oxide nanoparticles (SPIONs). A) Digital images of Col-I/BCf-SPIONs hydrogels and their proportions of Col-I and BCf-SPIONs. B) Digital images of top and lateral views of Col-I and Col-I/BCf-SPIONs hydrogels. Scale bars of 1 cm.

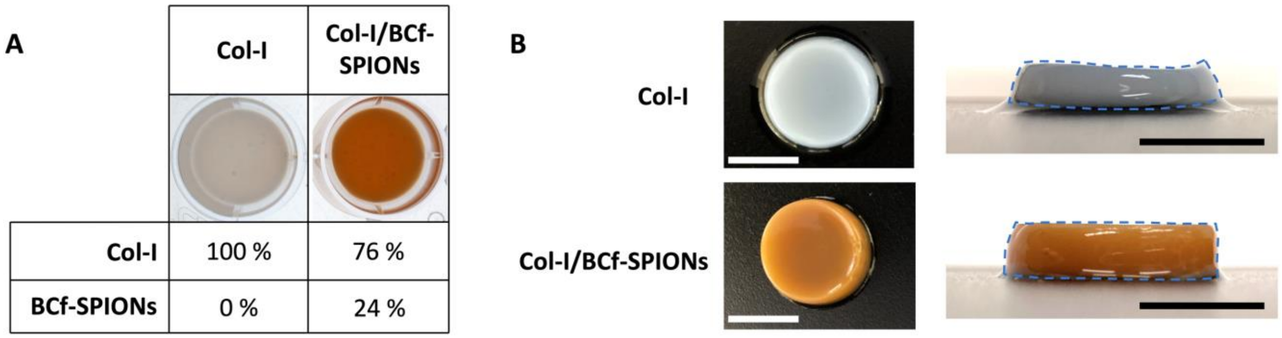

To confirm the homogenous distribution of BCf within the hydrogels, BCf were functionalized with iron oxide nanoparticles (SPIONs) before being mixed with the collagen. The orange color of the magnetic nanoparticles acted as sentinels to identify BCf at the macroscopic scale. The homogeneous coloration of those gels corroborated a homogeneous distribution of BCf within the hydrogel. Moreover, the composite exhibits straighter sides and a flatter surface than the pure Col-I hydrogel, which appears more collapsed (see the lateral view picture, Figure S1, panel B). Besides, in a future study, the magnetic particles could serve to actuate the gels by an external magnetic field.

Figure S2. Isotherms and pore size distribution plot obtained by Brunauer-Emmett-Teller (BET) analysis of Col-I/BCf and Col-I hydrogels. A) Isotherm of Col-I/BCf hydrogels. B) Isotherm of Col-I hydrogels. C) Desorption pore volume plot. Backgrounds from light to dark grey correspond to the micropores (< 2 nm), mesopores (2 - 50 nm), and macropores (> 50 nm) according to the IUPAC pores size classification[1].

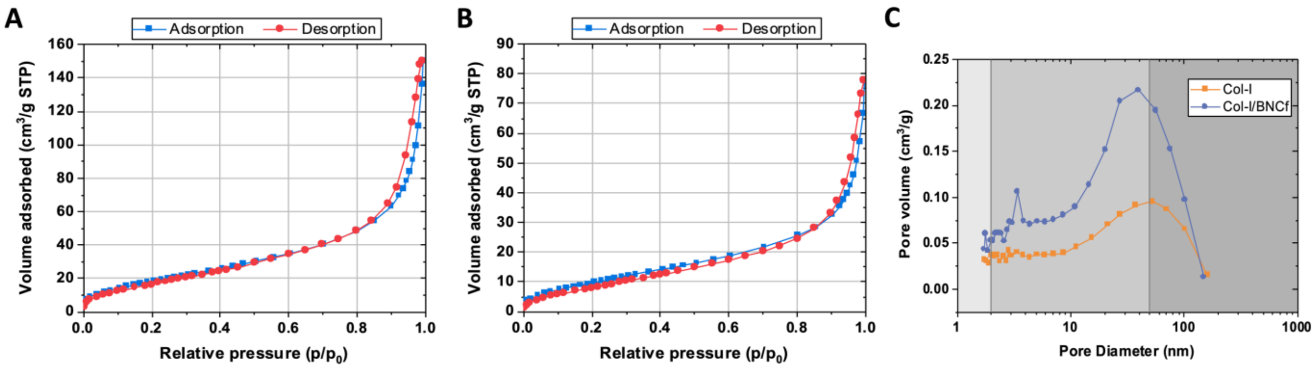

Table S1. Data from porosity analysis by nitrogen adsorption and desorption including mesopore average diameter and prevalence in Col-I hydrogels with and without BCf.

	**Col-I/BCf**	**Col-I**
Weight after degassing phase (g)	0.083	0.082
Equation of BET plot	y = 0.0587x + 0.0018	y = 0.1011x + 0.0055
Correlation coefficient	0.9994	0.9997
BET surface area (m^2/g)	72	41
BET constant (C)	33.0	19.4
BJH cumulative pore volume (cm^3/g)		
Adsorption	0.23	0.12
Desorption	0.23	0.12
BJH average pore diameter (4V/A) (nm)		
Adsorption	12	11
Desorption	11.30	11.17

Reference

1. Sing K. S. W.; Everett D. H.; Haul R. A. W.; Moscou L.; Robert A. Pierotti R. A.; Rouquerol J.; Siemieniewska T. Reporting physisorption data for gas/solid systems with special reference to the determination of surface area and porosity (Recommendations 1984). *Pure Appl Chem.* **1985**, *57* (4), 603-619. DOI: 10.1002/9783527610044.hetcat0065.
